# Identification and Characterization of a Novel Trehalose Synthase Gene Derived from Saline-Alkali Soil Metagenomes

**DOI:** 10.1371/journal.pone.0077437

**Published:** 2013-10-16

**Authors:** Ling Jiang, Ming Lin, Yang Zhang, Yanping Li, Xian Xu, Shuang Li, He Huang

**Affiliations:** 1 College of Food Science and Light Industry, Nanjing University of Technology, Nanjing, People’s Republic of China; 2 College of Biotechnology and Pharmaceutical Engineering, State Key Laboratory of Matericals-Oriented Chemical Engineering, Nanjing University of Technology, Nanjing, People’s Republic of China; 3 College of Sciences, Nanjing University of Technology, Nanjing, People’s Republic of China; University of Groningen, Netherlands

## Abstract

A novel trehalose synthase (TreS) gene was identified from a metagenomic library of saline-alkali soil by a simple activity-based screening system. Sequence analysis revealed that TreS encodes a protein of 552 amino acids, with a deduced molecular weight of 63.3 kDa. After being overexpressed in *Escherichia coli* and purified, the enzymatic properties of TreS were investigated. The recombinant TreS displayed its optimal activity at pH 9.0 and 45 °C, and the addition of most common metal ions (1 or 30 mM) had no inhibition effect on the enzymatic activity evidently, except for the divalent metal ions Zn^2+^ and Hg^2+^. Kinetic analysis showed that the recombinant TreS had a 4.1-fold higher catalytic efficientcy (*Kcat*/*K*_*m*_) for maltose than for trehalose. The maximum conversion rate of maltose into trehalose by the TreS was reached more than 78% at a relatively high maltose concentration (30%), making it a good candidate in the large-scale production of trehalsoe after further study. In addition, five amino acid residues, His172, Asp201, Glu251, His318 and Asp319, were shown to be conserved in the TreS, which were also important for glycosyl hydrolase family 13 enzyme catalysis.

## Introduction

Trehaolse is a naturally occurring non-reducing disaccharide in which the two glucose unites are linked via an α,α-(1,1)-glycosidic bond. Although there are three different anomers of trehalose (i.e. α,α-1,1-,α,β-1,1- and β,β-1,1-), the only known biologically active form is α,α-1,1-glucosyl-glucose [1]. This disaccharide has been isolated from a large number of prokaryotic and eukaryotic cells including mycobacteria, streptomycetes, enteric bacteria, archaea, yeast, fungi, algae, low orders of the animal kingdom and higher orders of the plant kingdom, especially those living in harsh environment [2,3]. Initially, trehalose was thought to act solely as a reserve energy and carbon source in a manner similar to that of starch and glycogen, but a growing number of studies indicate that this sugar instead has important biological function of playing a major role in cell protection against various physical and chemical stresses, such as heat, cold, dehydration, desiccation, oxygen radicals, and acidic/alkali environmental conditions [4-6]. Moreover, the ability of the microorganisms to survive in these environments correlates with their trehalose content [7,8]. In yeast, the most ancient actor of biotechnological transformation, trehalose was found to accumulate, in certain physiological conditions, up to 10%-15% of cell dry weight [9]. Investigation on the cell membranes of anhydrobiotic yeast has unraveled that intracellular trehalose can stabilize proteins in their native state and to reduce their heat-induced denaturation and aggregation [10,11]. As a matter of fact, the build-up of trehalose upon heat shock has been shown to be more important than the induction of heat-shock proteins as a thermotolerance response element [12]. Also, trehalose was shown to decrease oxidative damage to cell proteins by oxygen radicals and thus to increase the tolerance of organisms to reactive oxygen species [13]. In particular, trehalose also has the same protection effect *in vitro*, which opens a new field for its application in food, cosmetic, and pharmaceutical industries, ranging from serving as a sweetener to a biomaterial stabilizer [14]. Besides acting as a protectant, trehalose is also an important component of the cell walls of many mycobacteria and corynebacteria in the form of glycolipids. A well-known example is trehalose dimycolate (or cord factor), which was composed by a trehalose core with mycolic acid esterified at the 6-OH and 6'-OH positions [15]. The cord factor is the most toxic lipid produced by *Mycobacterium tuberculosis* and dramatically increases the impermeability of the cell wall to various antibiotics and thus was identified as a virulence factor [16]. 

Although its usefulness was widespreadly recognized, the cost of commercialized trehalose could reach as high as seven hundred US$·kg^-1^ in the early 1990s, which was not compatible with emerging applications [17]. The conventional method for production, for example, extraction from transformed plants, as will as fermentation of yeast and *Corynebacterium*, had too low a yield and too high a cost to be used. In 1995, the Hayashibara Co. Ltd. has isolated a two-step enzymatic system from a bacterial strain belonging to the genus *Arthrobacter* sp. Q36 which was obtained from soil [18]. The novel approach of trehalose production had led to a major reduction in the commercial price of trehalose to 5-6 US$·kg^-1^, and for the first time, successfully exploited in industrial production of trehalose. However, further decrease the production cost of trehalose could be achieved no other than the application of the brand-new enzymatic route [19].

Trehalose synthase (TreS, EC 5.4.99.16) was first demonstrated in *Pimelobacter* sp. R48 through an extensive screening of 2,500 strains of soil bacteria [20]. It can catalyze the intramolecular rearrangement of maltose into trehalose in a single step, which represented a simple, fast, and low-cost method for the future industrial production of trehalose [1]. So far, a number of TreSs from several bacterial strains have been identified and characterized (Table 1). However, these TreSs were still not satisfying in a practical application with regard to either their activities or conversion efficiency. Furthermore, all these TreSs were from cultured microorganisms, and little attention had been paid to those from uncultivable microorganisms, which may account for more than 99% of microorganisms in the environment [21]. It is imaginable that there is a large number of industry-potential TreSs for the production of trehalose in the uncultivable microorganisms of environment.

**Table 1:** Summary of several identified and characterized TreSs from bacterial strains.

Strain name	Gene size (bp)	Molecular size (kDa)	Optimum temperature (℃)	Optimum pH	Optimum substrate	Conversion rate	Citation
*Mycobacterium smegmatis* ATCC 14468	1781	71	37	7.2	0.5mM	45%	Pan et al., 2004
*Thermobifida fusca* DSM 43792	1830	66	25	6.5	800mM	60%	Wei et al., 2004
*Pseudomonas stutzeri* CJ 38	2070	76	35	8.5	600mM	72%	Lee et al., 2005
*Picrophilus torridus* DSM 9790	1677	65	45	6	150mM	71%	Chen et al., 2006
*Thermus thermophilus* ATCC 33923	2898	106	65	6.5	3mM	62%	Wang et al., 2007; Anna et al., 2005
*Deinococcus radiodurans* ATCC 13939	1659	61	15	6.5	800mM	65%	Wang et al., 2007; Wei et al., 2004
*Arthrobacter aurescens* CGMCC1.1892	1797	68	35	6.5	90mM	60%	Wu et al., 2009
*Enterobacter hormaechei*	1626	65	37	6	100mM	48%	Yue et al., 2009
*Meiothermus ruber* CBS-01	2889	106	50	6.5	60mM	65%	Zhu et al., 2010
*Corynebacterium glutamicum* ATCC 13032	1812	70	35	7	3mM	69%	Kim et al., 2010
*Deinococcus geothermalis* DSMZ 11300	1692	65	40	7.6	300mM	60%	Pawel et al., 2012
*Thermomonospora curvata* DSM 431383	1806	60	35	6.5	3mM	70%	Liang et al., 2013
*Rhodococcus opacus*ATCC 41021	1857	79	25	7	90mM	67%	Yan et al., 2013
*Deinococcus* sp.	1656	63.3	45	9	800mM	78%	This study

To expand the range of TreSs discovery, culture-based methods have been complemented or replaced by culture-independent metagenomic approaches, which theoretically provide access to the collective nucleic acids from the uncultivable organisms of various environmental samples [22]. Functional metagenomics based on the direct isolation of DNA from soil environmental samples, generation of metagenomic libraries from the isolated DNA, and function-driven screening of the constructed libraries has been successfully employed in the identification and characterization of enzymes with special biocatalytic activities [23]. In the present study, a metagenomic library from saline-alkali soil sample of Lop Nur in Xinjiang Uigur Autonomous Region of north-west China was constructed for the screening new TreSs. Finally, one novel TreS with high activity and conversion efficiency was identified and subsequently expressed in *Escherichia coli* (*E. coli*). The specific enzymatic properties of the recombinant enzyme were also characterized after purification. Furthermore, the functional amino acid residues have been predicted by the site directed mutation based on homology modeling and structure analysis.

## Materials and Methods

### Bacterial strains and cultivation

*E. coli* DH5α was used for construction of recombinant plasmids and *E. coli* BL21(DE3) was used as expression host. *Thermus thermophilus* ATCC 33923 was purchased from the China General Microbiological Culture Collection Center (CGMCC). The pUC118 (TaKaRa, Dalian, China) and pET-22b(+) were used to construct metagenomic libraries and express the target protein, respectively. *E. coli* transformants were grown at 37 °C in Luria-Bertani medium containing 50 μg·mL^-1^ ampicillin [24].

### Isolation of DNA from environmental sample

For the construction of a metagenomic library, an environmental sample was obtained from the soil in Lop Nur. The total DNA was extracted based on a method described previously [25]. Soil sample (4 g of wet weight) was mixed with 13.5 mL of DNA extraction buffer, which composed of 100 mM Tris-HCl (pH 8.0), 100 mM sodium EDTA (pH 8.0), 100 mM sodium phosphate (pH 8.0), 1.5 M NaCl, 1% cetyltrimethylammonium bromide (CTAB), and 1.5 mL of 20% sodium dodecyl sulfate (SDS). The mixture was incubated in a 65 °C water bath for 2 h with gentle inversion every 15 to 20 min. The supernatants were collected after centrifugation (6,000 × g, 10 min) at room temperature and transferred into 50 mL centrifuge tubes. An equal volume of chloroform/isoamylol (24:1) was added and gently mixed. The aqueous phase was recovered by centrifugation and precipitated with 0.6 volume of isopropanol at room temperature for 1 h. The crude nucleic acids was obtained by centrifugation (16,000 × g, 20 min) at 4 °C, washed twice with cold 70% ethanol and suspended in an appropriate volume of sterile deionized water.

### Construction of a metagenomic library

To construct the metagenomic library, the purified DNA was partially digested with BamHI. DNA fragments of 2.5-10 kb were ligated into BamHI-digested pUC118, and the ligated products were transformed into *E. coli* DH5α. The transformed cells were plated onto LB agar plates containing 50 μg/mL ampicillin (Amp), 0.5 mM isopropyl-β-D-thiogalactopyranoside (IPTG) and 100 μM 5-Bromo-4-chloro-3-indolyl β-D-galactopyranoside (X-gal). After incubation at 37 °C for 24 h, clones with white color were selected and further tested by colony polymerase chain reaction (PCR). 

### Subcloning and gene sequence analysis

**Figure 1:**
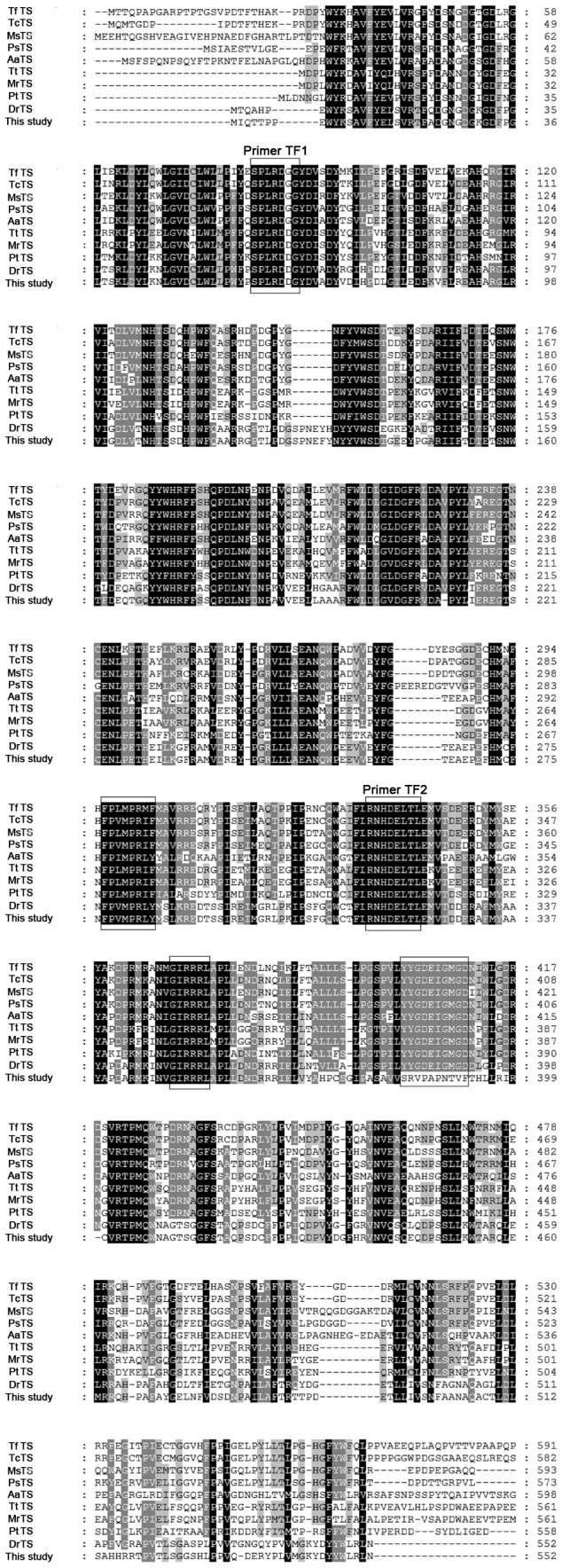
Multiple sequence alignment of trehalose synthase. TfTS, *Thermobifida fusca* TreS (AAZ54622.1); TcTS, *Thermomonospora curvata* TreS(ACY99119.1); MsTS, *Mycobacterium smegmatis* TreS(ABK71531.1); PsTS, *Pimelobacter* sp. TreS(BAA11303.1); AaTS, *Arthrobacter* *aurescens* TreS(ACL80570.1); TtTS, *Thermus* *thermophilus* TreS(BAA19934.1); MrTS, *Meiothermus* *ruber* TreS(YP003508484); PtTS, *Picrophilus* *torridus* TreS(YP022847.1); DrTS, *Deinococcus radiodurans* TreS(ACL80570.1). Amino acid residues that are identical are shaded in black boxes, whereas conserved residues are shaded in gray boxes. The dashed spaces represent gaps to maximize alignment.

Several bacterial trehalose synthase sequences published in the NCBI database were collected and analyzed by the online multiple sequence alignment program CLUSTAL W2 (http://www.ebi.ac.uk/Tools/clustalw2). As shown in Figure 1, two degenerate primers TF1 (5’-AGYCCNCTNCGNGAYGRNGGNT-3’) and TF2 (5’-AGNGTNAGYTCRTCRTGRTT-3’) were synthesized based on the conserved domains. Then colony PCR was carried with primer TF1 and TF2, clones in white color were used as templates, the genome of *Thermus thermophilus* ATCC 33923 was acted as a positive control. PCR products were detected on a 2% agarose gel. Only the clones containing recombinant plasmids (pUC118-*treS*) as well as positive control could produce detectable band. Then the recombinant plasmids were extracted and sequenced. The deduced amino acid sequence analysis and open reading frame search were performed with BLAST program provided by NCBI (http://www.ncbi.nlm.nih.gov/). The phylogenetic tree was constructed by the neighbor-joining methodusing Molecular Evolutionary Genetics Analysis software (MEGA, version3.1).

### Cloning of the *treS* gene and construction of the expression vector

Primers TR1 (5’-CCCATATGATGATCCAGACCACCCCACCAG-3’) and TR2 (5’-CCAAGCTTGTTCAGGCGCAGCCAGTAATAGT-3’) were synthesized according to the open reading frame (ORF) sequence to introduce NdeІ and HindⅢ sites into the 3' and 5' ends of *treS* ORF, respectively. The stop condon of *treS* was eliminated to in-frame read a His(6)-tag on the C-terminus for one step purification. The *treS* gene was amplified by PCR with the pUC118-*treS* as template. The PCR product was then digested with restriction enzymes NdeІ and HindⅢ, and inserted into pET22b(+) vector to generate pET22b(+)-*treS*. After confirmed by DNA sequencing, the recombinant plasmid was transformed to the *E. coli* BL21(DE3). 

### Protein expression and purification

The *E. coli* BL21(DE3) harboring pET22b(+)-*treS* was inoculated into LB medium supplemented with 50 μg·mL^-1^ Amp and then grown at 37 °C in a shaker at 200 rpm. When the OD_600_ of the culture reached 0.8, IPTG was added to a final concentration of 1.0 mM, and then the incubatin was continued for another 8 h at 30 °C. The cells were harvested by centrifugation and resuspended in lysis buffer (50 mM KH_2_PO_4_-K_2_HPO_4_, 500 mM NaCl, pH 6.0) followed by sonification and centrifugation at 12,000 × g for 20 min at 4 °C to remove insoluble cell debris. The supernatant was filtered through a 0.45-μm filter and loaded onto a Ni-NTA affinity chromatography column according to the manufacturer’s purification protocol manual (Novagen, Ni-NTA His·Bind Resins). The cell extracts and purified enzyme were analyzed by 12.5% (w/w) SDS-polyacrylamide gel electrophoresis (SDS-PAGE). Protein concentrations were determined by the method of Bradford using bovine serum albumin as a standard [26].

### Activity assay of recombinant TreS

The activity of TreS was assayed by measuring the amount of trehalose produced from maltose. The reaction was performed in a mixture containing the TreS solution and 100 mM maltose in 50 mM phosphate buffer (pH 9.0) at 45 °C for 30 min, then boiled for 10 min to terminate the reaction. The amount of trehalose, glucose, and maltose of each reaction mixture was measured using a high-performance liquid chromatography (HPLC) system equipped with an RID (Shodex, China) detector at a flow rate of 0.9 mL·min^-1^. A NH_2_ column (Sepax, US) equilibrated with 75% acetonitrile, 25% Milli-Q water was used. The retention times of glucose, maltose, and trehalose were 8.0, 11.2, and 12.5 min, respectively. One unit (U) of enzyme activity was defined as the amount of enzyme that catalyzes the formation of 1 μmol trehalose per min under the specified conditions. The conversion rate was calculated by the ratio of the trehalose product to the amount of maltose substrate.

### Properties of recombinant TreS

The optimum pH of TreS was assayed by incubating the purified enzyme with 200 mM maltose substrate in 50 mM potassium phosphate buffer at pH 3.0 to 11.0 and 45 °C for 30 min, respectively. The optimum temperature for TreS activity was determined at 10 to 80 °C using the same buffer at pH 9.0 for 30 min, respectively. To determine the effect of metal ions and different chemical reagents on TreS, its activity was also assayed in the presence of these ions or compounds at 1 mM, respectively. To determine the conversion efficiency of TreS, the 5-L reaction system consisted of a 5-L stirred-tank fermentor (B. Braun, B. Braun Biotech International, Melsungen, Germany) containing 2-L of a medium was employed [27].

### Determination of kinetic parameters

The Michaelis-Menten (*K*_*m*_) and maximum activity (*V*_*max*_) constant for recombinant TreS were determined under conditions of pH 9.0 and 45 °C for 30 min in 200 mM sodium phosphate buffer containing substrate (maltose and trehalose) at various concentrations. The resulting data were plotted with Origin 6.0 software (Microcal, Northampton, MA). All experiments were carried out in triplicate. 

### Construction and analysis of protein models for TreS

Models were built through an online Automatic Modeling Mode server at http://swissmodel.expasy.org. Obtained models were analyzed through Swiss-Pdb Viewer [28] and PyMOL (http://www.pymol.org). Structure predictions for TreS were made by the development of the homology model using the resolved X-ray structure of α-amylase with Protein database entry code 1SMA as template.

### Site-directed mutagenesis of TreS

Mutants were obtained through a cloning method with two complementary primers containing mutation bases. The pET22b(+)-*treS* plasmids were ampliﬁed by PCR with PrimeSTAR HS DNA polymerase (Takara, Dalian, China). To remove the templates, DpnІ was added to PCR reactions for 1 h at 37 °C. The digested products were then directly transformed into competent *E. coli* DH5α to obtain mutation recombinant plasmids. After identiﬁed by sequencing, the recombinant plasmid containing mutation site was transformed to the *E. coli* BL21(DE3).

## Results

### Construction and screening of the metagenomic library

A metagenomic library of *ca*. 85,000 clones was constructed for obtaining trehalose synthase genes. Restriction analysis of 20 randomly selected clones indicated that all the clones harbored insertion DNAs ranging from 2.5 to 5 kb in size, with an average of approximating 3.5 kb. The metagenomic library processed a capacity ~300 Mb of soil microbial genomic DNA. One clone expressing trehalose synthase activity was isolated after all the clones in the library had been screened. 

### Sequence analysis of *treS* gene

**Figure 2:**
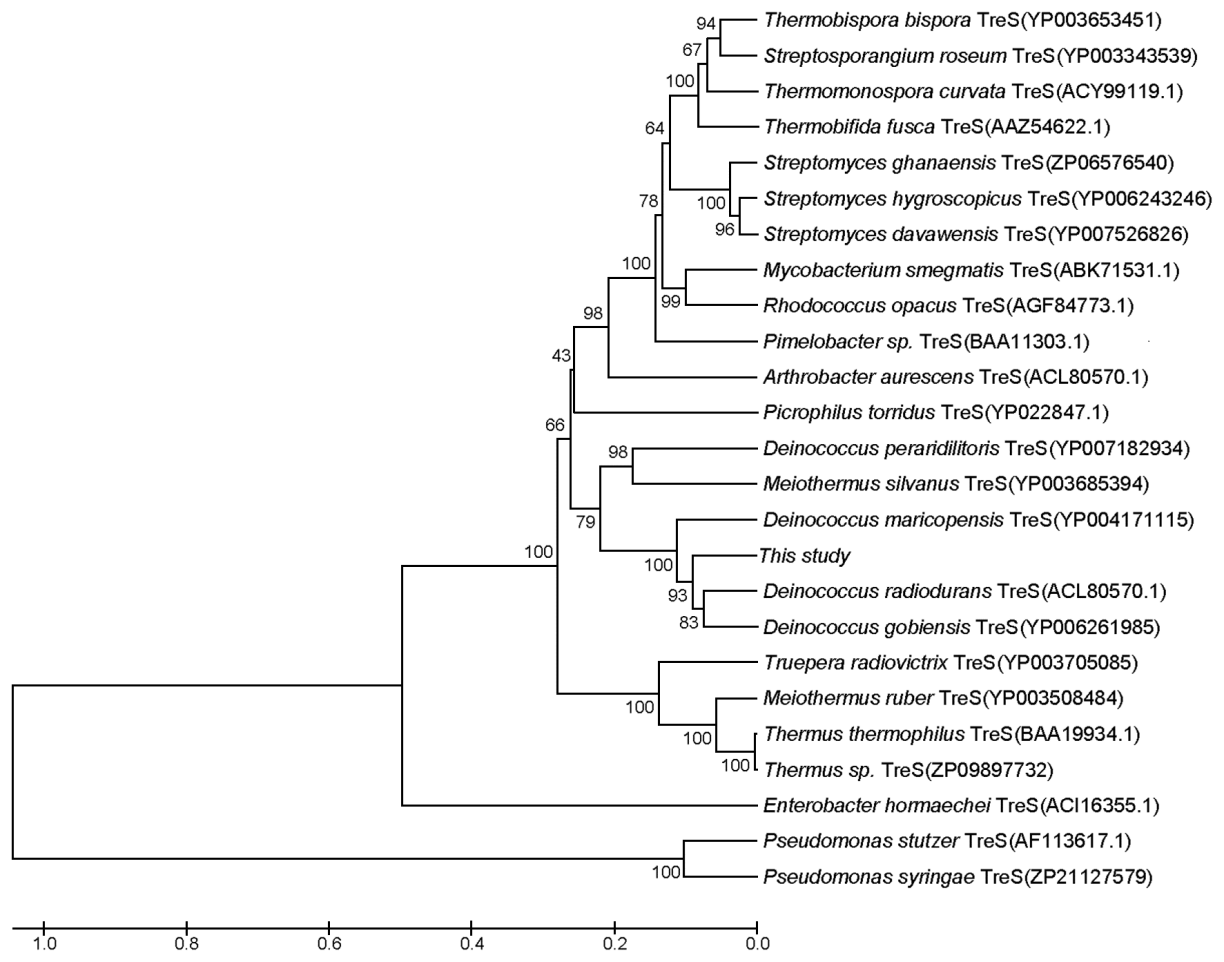
Phylogenetic tree analysis of TreS in this study and other trehalose synthases.

The plasmid of pUC118-*treS* was extracted and the complete insert DNA was sequenced. The length of the insert DNA was 5,356 bp. An ORF-ﬁnder and BLAST analysis revealed the presence of an ORF consisting of 1,656 bp, encoding a full-length *treS* gene, which further encoded a protein of 552 amino acids with a predicted molecular mass of 63.3 kDa. The deduced amino acid sequence of *treS* was used to perform a BLAST research of the NCBI and SwissProt databases. This search revealed that the protein has the highest similarity (84%) with trehalose synthase from *Deinococcus radiodurans* R1 [29]. As can be seen in Figure 1, multiple sequence alignments of this *treS* gene with 10 reported trehalose synthase revealed that they share several highly conserved amino acid motifs, such as SPLRDG/DGYDV/I, FPL/VMPRI/LF/Y, NHDELTLE, GIRRRLA/MPL, and so on. However, the region SRVPAPNTVF in this *treS* gene of the present study was significantly different from the conserved regions YYGDEIGMGD in other listed *treS* genes (Figure 1). Such a result suggests that the cloned fragment may originate from an uncultured organism, and the identified gene-encoding products possibly had a unique function. The phylogenetic tree based on amino acid sequence was further constructed to verify the evolutionary relationship of this *treS* gene to other known trehalose synthases, and 24 trehalose synthase proteins were selected for the phylogenetic tree analysis. As shown in Figure 2, this recombinant TreS has a close relationship to *Deinococcus* genus regarding sequence homology.

### Expression and purification of the recombinant TreS

**Figure 3:**
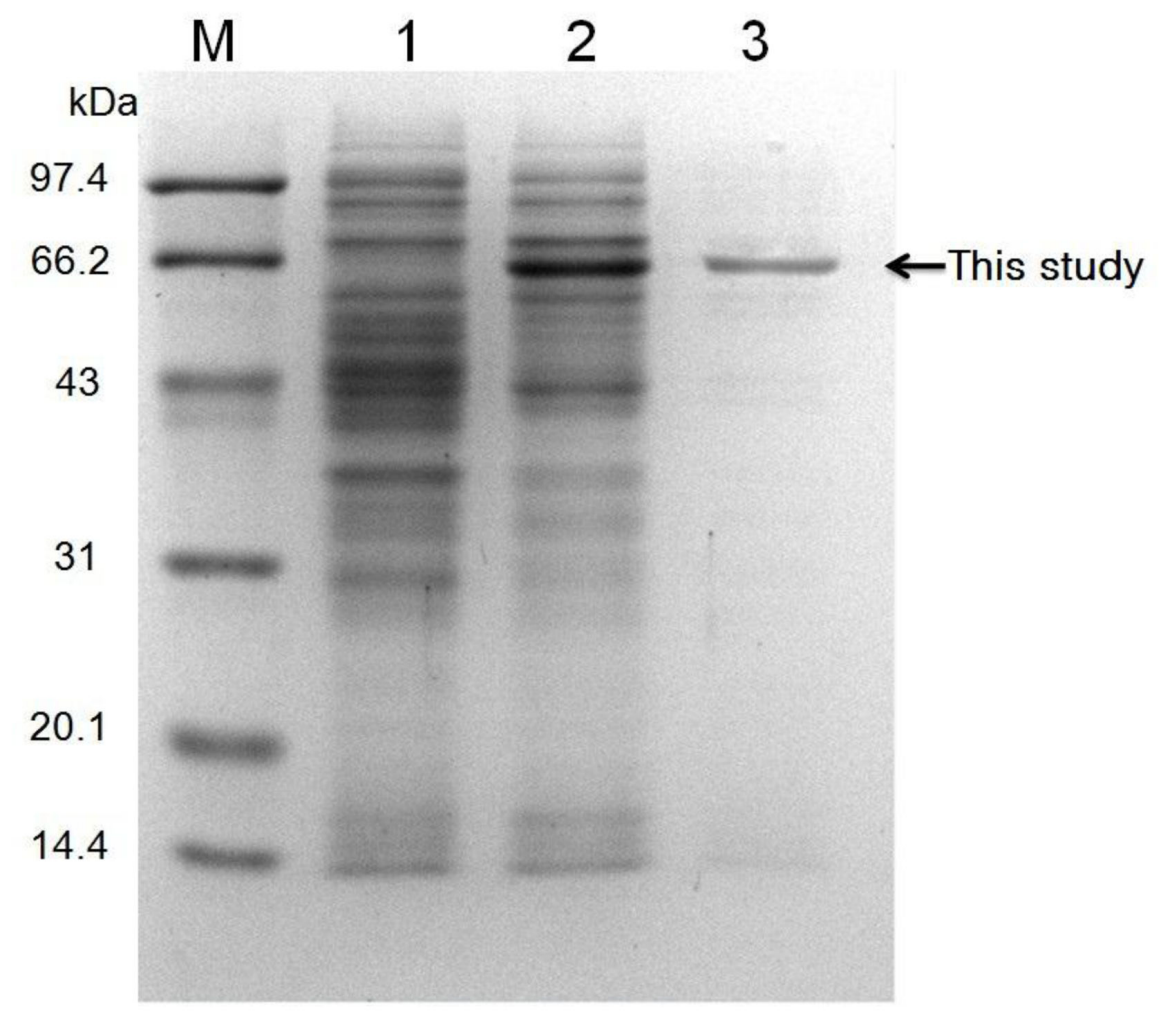
SDS-PAGE analysis of the puriﬁed recombinant TreS. Lane M is protein molecular weight markers. Lane 1 is the crude extract of the recombinant strain *E. coli* BL21 with pET22b. Lane 2 is the crude extract of the recombinant strain *E. coli* BL21 with pET22b-*treS*. Lane 3 is the recombinant enzyme TreS purified using Ni-NTA affinity chromatography. The arrow indicates the recombinant TreS in this study.

To characterize the biochemical properties of the recombinant TreS, the *treS* gene was expressed as an N-terminal His-tag fusion protein using pET22b(+) expression system in *E. coli* BL21(DE3). No inclusion bodies were found in cell lysates, which suggested that this recombinant TreS was expressed in a soluble form. The cells were harvested and disrupted by sonication on ice. When compared to the sample without induction (Figure 3, lanes 1), only the induced cells containing the recombinant vector expressed an extra protein band migrating at about 65 kDa upon induction (Figure 3, lanes 2). The recombinant protein was about 1.5 kDa heavier than the predicted molecular mass of 63.3 kDa, which was due to the additional 13 amino acids including the His(6)-tag at the N-terminus. After purification with the Ni-NTA column, a single band was shown on the SDS-PAGE gel correlating with the size of enzyme, indicating that the recombinant enzyme was purified to homogeneity (Figure 3, lane 3).

### Effects of pH and temperature on activity of TreS

**Figure 4:**
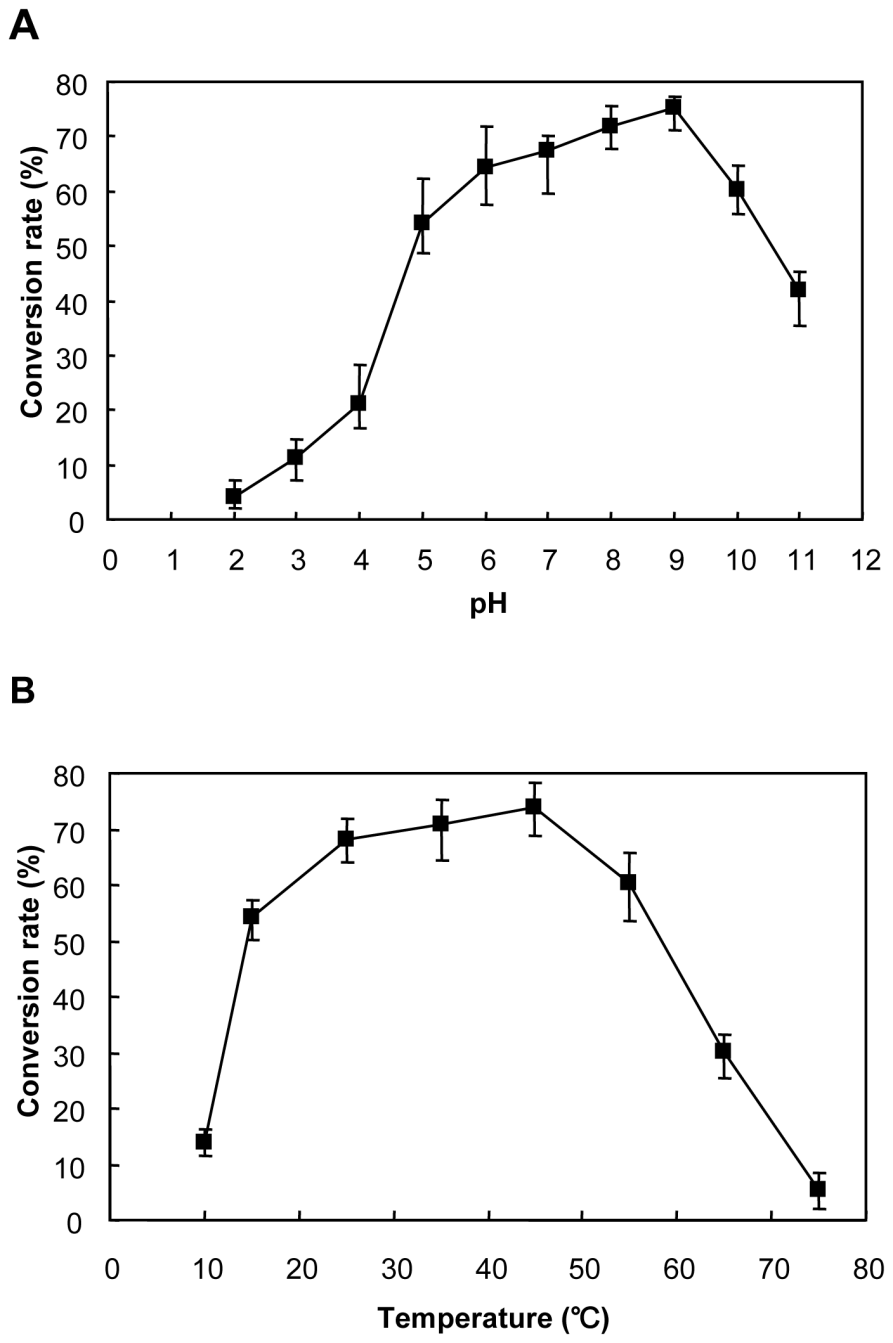
Effects of pH (A) and temperature (B) on the activity of TreS. (A) The enzyme activity at various pH values were examined at the maltose concentration of 200 mM and 45 °C for 30 min. (B) The enzyme activity at various temperature were examined at the maltose concentration of 200 mM and pH 9.0 for 30 min. The average of triplicate experiments is presented.

The optimum pH of the recombinant TreS was found to be 9.0. The enzyme maintained high activity at a broad pH range of 5.0-10.0 (Figure 4A). The optimum temperature was 45 °C. Meanwhile, the enzyme maintained high activity when reaction temperature ranged from 15 °C to 55 °C. However, relative activity quickly decreased when temperature was above 55 °C or below 15 °C (Figure 4B). Thus, it is probably more efficient to carry out conversions at moderate temperatures. In this study, the recombinant TreS exhibited a stable performance under the wide working conditions (pH 5.0-10.0 and 15-55 °C, Figure 4). Further studies of the effects of temperature, reaction mixtures containing 200 mM maltose were incubated under pH 9.0 at 55 °C for 1 h, the remaining activity of the recombinant TreS was as high as 80% of its initial activity.

### Activity assay of the recombinant TreS

Enzymatic activity was detected with purified TreS in reactions of the conversion between maltose and trehalose. It was confirmed that the DNA fragment was the intrinsically coding sequence of active TreS. The highest enzyme activity was calculated to be 133.5 ± 4.8 U·mg^-1^ protein, and the conversion efficiency was test in a 5-L reaction system. After 18 h of TreS-catalyzed reaction, the final yield of trehalose was constantly above 75%, with the maximum value of 78% under the optimum condition at a relatively high maltose concentration (30%).

### Effects of mental ions and reagents on activity of TreS

The effects of metal ions and reagents were further determined by examining recombinant TreS activity in the presence of 1 and 30 mM of these substances under standard assay conditions (Table 2). As shown in Table 2, the TreS activity was inhibited strongly by Hg^2+^, Zn^2+^, and SDS and moderately by Cu^2+^, Cd^2+^, Pb^2+^, and Tris at the concentration of 1 mM. More uplifting was the result that, at the concentration of 30 mM, almost all metal ions and reagents, except for Hg^2+^ and Zn^2+^, had no more inhibition effect on the enzyme activity than the concentration of 1 mM.

**Table 2:** Effects of metal ions and reagents on the activity of TreS.

Reagent	Relative activity^a^ (%)		Reagent	Relative activity^a^ (%)	
	1 mM	30 mM		1 mM	30 mM
none	100	100	MgCl_2_	106 ± 6	104 ± 3
ZnSO_4_	69 ± 4	41 ± 5	MnCl_2_	99 ± 5	98 ± 6
CuSO_4_	82 ± 6	80 ± 4	BaCl_2_	98 ± 2	95 ± 5
CdSO_4_	78 ± 5	74 ± 5	CaCl_2_	97 ± 6	98 ± 5
Al_2_(SO_4_)_3_	104 ± 5	103 ± 2	PbCl_2_	82 ± 5	78 ± 3
FeSO_4_	99 ± 4	98 ± 5	HgCl_2_	68 ± 5	34 ± 3
DTT	98 ± 3	85 ± 5	SrCl_2_	95 ± 7	92 ± 2
EDTA	97 ± 2	93 ± 4	NiCl_2_	90 ± 4	88 ± 5
SDS	65 ± 5	58 ± 3	CoCl_2_	93 ± 1	91 ± 3
Tris	74 ± 2	65 ± 5			

^a^ Enzyme activity was measured in the presence of 1 and 30 mM metal ions or reagents under assay conditions of temperature 45 °C, pH 9.0, and 200 mM maltose for 30 min. Relative activity is expressed as a percentage of the enzyme activity in the absence of metal ions and reagents. The average of triplicate experiments is presented.

### Kinetic analysis of TreS

Kinetic parameters of the recombinant TreS were investigated at pH 9.0 and 45 °C for 30 min with maltose or trehalose as substrate. When this data was plotted by the method of Lineweaver and Burk, the *K*_*m*_ values for the recombinant TreS were found to be 9 ±1.2 mM for maltose and 64 ± 3.5 mM for trehalose, the *V*_*max*_ values of 1.5 ± 0.2 mM·min^-1^ mg^-1^ protein for maltose and 3.1 ± 0.6 mM·min^-1^ mg^-1^ protein for trehalose were calculated, respectively. Although a higher *K*_*cat*_ for trehalose than maltose was seen (78 ± 6.5 s^-1^ vs. 44 ± 3.8 s^-1^), TreS had a 4.1-fold higher catalytic efficiency (*Kcat*/*K*_*m*_) toward maltose than trehalose (4.9 ± 1.2 M^-1^ s^-1^ vs. 1.2 ± 0.18 M^-1^ s^-1^). With regard to these results, the recombinant TreS had a higher affinity to maltose and a favorite reaction direction toward the synthesis of trehalose. Interestingly, all reported TreS enzymes share the feature of a reversible conversion at different degrees [26,29]. 

### Structure analysis and site-directed mutagenesis

**Figure 5:**
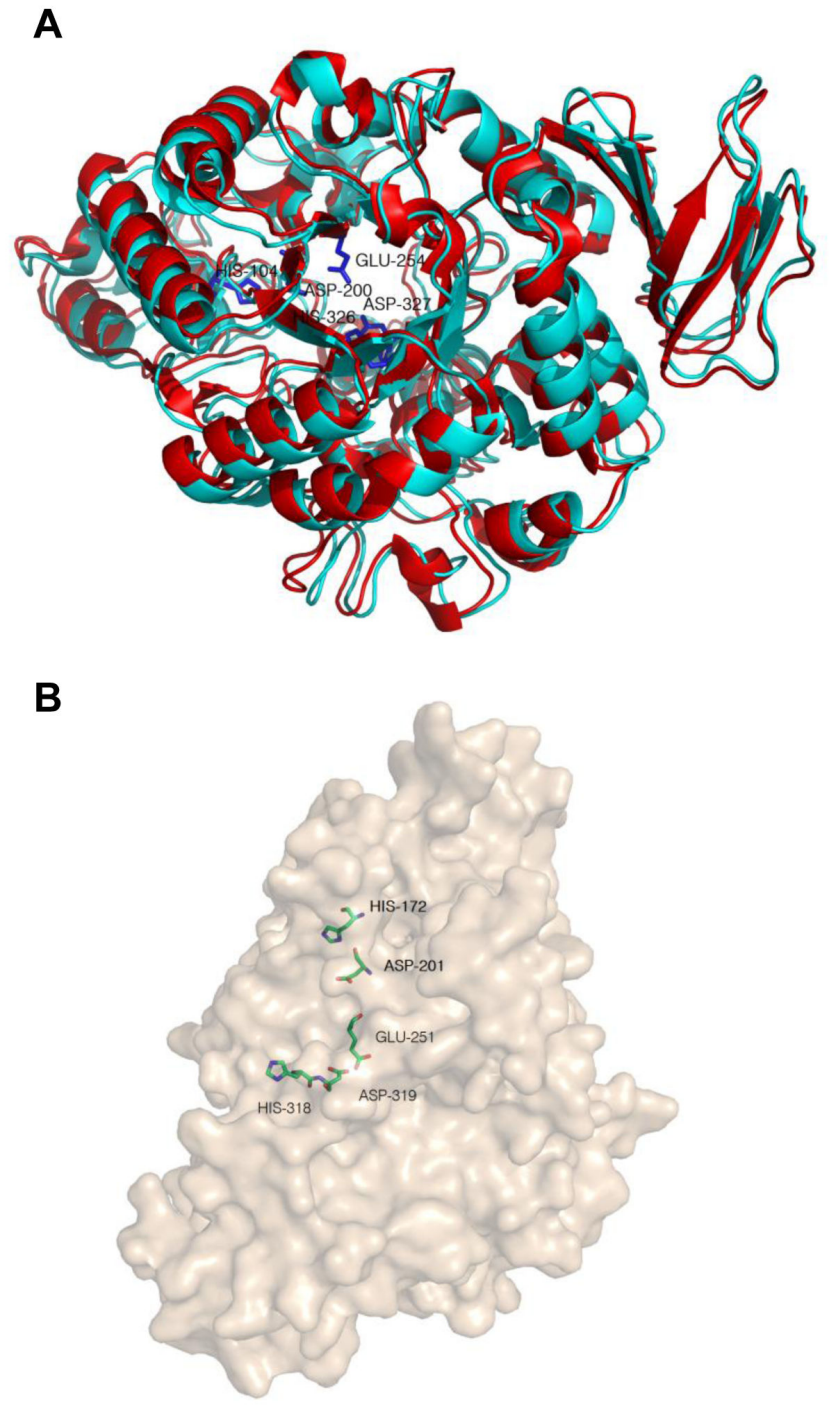
Comparative modeling of TreS based on known trehalulose synthase template. (A) Model of TreS (blue) was superimposed with 2PWG (red). Five key amino acids (His104, Asp200, Glu254, His326, and Asp327) in the active center of 2PWG are labeled with marked sticks inside the (α/β)8 barrel catalytic domain. (B) Side view of the surface model for TreS and its five conserved amino acids (His172, Asp201, Glu251, His318, and Asp319) in the active center. The key amino acids are indicated by sticks and name of residue.

Using the structure-resolved *Pseudomonas* Mesoacidophila trehalulose synthase as template (PDB ID: 2PWG), a TreS model was built through SWISS-MODEL. The sequence identity of TreS and trehalulose synthase was 30%, but both belonged to the glycosyl hydrolase family 13 (GH13 family) and had a typical (α/β)_8_ barrel catalytic domain (Figure 5A). In alignment with the amino acid sequences of trehalulose synthase, five conserved key amino acids constituting a catalytic pocket (His172, Asp201, Glu251, His318 and Asp319) of TreS were deduced. Three dimensional structures showed that all five putative active sites were in the center of the barrel catalytic domain (Figure 5B). To verify the importance of these residues, site-directed mutagenesis was used to replace the five residues individually with Ala, and each mutant recombinant protein was purified by Ni column. The drastic reduction in enzyme activity of all mutants suggested that these five residues might play essential roles in TreS catalysis (Table 3). Moreover, similar conservations of active sites were observed in other TreSs (Figure 1), further supporting the catalytic importance of these residues in TreS activity.

**Table 3:** Relative specific activity of wild-type TreS and mutant enzymes.

mutant	relative specific activity^a^ (%)	mutant	relative specific activity^a^ (%)
wide type	100.00	E251A	1.71 ± 0.12
H172A	7.21 ± 0.51	H318A	0.00
D201A	4.76 ± 0.24	D319A	0.00

a Relative activities are represented as the ratio of mutants to wild type. The specific activity of wild-type TreS was 133 units/mg protein.

## Discussion

Up to now, five different enzymatic routes involved in the biosynthesis of trehalose have been discovered and indentified from all kinds of environmental microorganisms. The first pathway utilizes trehalose-phosphate synthase (TPS, EC 2.4.1.15) (OtsA in *E. coli*) that catalyzes the transfer of the glucosyl moiety from UDP-glucose to glucose-1-phosphate, forming the intermediate trehalose-6-phosphate and UDP. The phosphate is then removed by trehalose-phosphate phosphatase (TPP, EC 3.1.3.12) (OtsB in *E. coli*) to give free trehalose [30]. The second pathway involves a trehalose glycosyltransferring synthase (TreT, EC 2.4.1.245) that catalyzes the synthesis of trehalose using nucleoside diphosphate glucose (NDPG), such as UDPG, as a donor and glucose as an acceptor [31]. The third pathway utilizes trehalose phosphorylase (TreP, EC 2.4.1.64) to catalyze a reversible reaction in which it hydrolyzes trehalose in the presence of inorganic phosphate to form glucose-1-phosphate and glucose and, inversely, gives rise to trehalose from both products *in vitro* [32]. However, all these three pathways are not suitable in trehalose industrial production due to their expensive substrates. The fourth pathway also involves a two-step enzymatic process, which utilizes glycogen or maltooligosaccharides as the starting material. The maltosyl moiety at the reducing end is first isomerized into a trehalosyl moiety by maltooligosyl trehalose synthase (TreY, EC 5.4.99.15). Hydrolytic release of the trehalose is then catalyzed by maltooligosyltrehalose trehalohydrolase (TreZ, EC 3.2.1.141) [33]. Trehalose has mainly been manufactured through this pathway since it was discovered in 1995. The last pathway utilizes only one enzyme, trehalose synthase (TreS, EC 5.4.99.16), to catalyze the intramolecular rearrangement of the α-1,4-linkage of maltose to the α-1,1-linkage of trehalose [34]. Maltose is relatively cheap, and this single step process saves both time and costs in scale-up mode, which could be an alternative method for industrial trehalose production.

In the present study, we constructed a plasmid metagenomic library from uncultivated microorganisms within saline-alkali soil samples and isolated a novel TreS gene by a sequence-based screening strategy. This recombinant TreS showed an optimal pH of 9.0 and an optimal temperature of 45 °C and retained 80% of its initial activity after heat-treatment at 55 °C for 1 h, which was consistent with the extreme environment such as high temperature, high salinity conditions in Lop Nur region. In comparison with other previously reported trehalose synthases, this recombinant TreS has the most alkali optimum pH. Moreover, the three well-characterized TreSs from *Thermobifida fusca*, *Enterobacter hormachei*, *Arthrobacter aurescens* showed dramatic decreases in enzyme activity at pH above 9.0 and retained <40% of activity [35-37]. Therefore, it is more alkali-tolerant than other TreSs reported so far. Using an enzyme that is stable at high pH value as well as high reaction temperature can reduce the possibility of microbial contamination, lower viscosity, and improve the solubility of substrates for better access to enzymatic attack. 

It is well known that a glycosidase activity is frequently affected by the presence of metal ions [38]. Various divalent metal ions were therefore examined and consequently found to have inhibition effect on the enzyme activity in different degree. The results showed that the activity of the recombinant TreS was decreased by Zn^2+^ and Hg^2+^, but not as significantly as other known TreSs, especially at a high concentration of the metal ions (up to 30 mM) [39,40]. However, the activity of this recombinant TreS was not affected by EDTA, suggesting that this enzyme did not require metal ions for activity and stability. 

To the target substrate maltose, the specific activity of this recombinant TreS was estimated to be about 133 units/mg of protein, which was 1.67-fold higher than that of *Pseudomonas stutzeri* and *Picrophilus torridus* trehalose synthases, and roughly equal to that of *Thermus aquaticus* trehalose synthase [20,39,41]. Because TreS catalyzes the interconversion of maltose and trehalose, but converts maltose to trehalose more rapidly than trehalose to maltose, it was therefore of interest to determine the affinity (*K*_*m*_) of this recombinant TreS for these two substrates. The results showed that the TreS had much greater affinity (7.1-fold) for maltose than trehalose. Moreover, the catalytic efficientcy (*Kcat*/*K*_*m*_) for maltose was 4.1-fold higher than trehalose, due to the reduced *K*_*m*_ value as well as the increased *K*_*cat*_ value, which resulted in the conversion of trehalose. In this study, the maximum trehalose yield from maltose by this recombinant TreS was exceeded 78% under the optimum condition, which was close to the theoretical equilibrium constant for overall reaction of 82% in favor of trehalose using thermodynamic parameters [42]. 

Although no three-dimensional structures have been obtained to date on trehalose synthase, previous reports on TreS have indicated that this enzyme was belonged to the GH13 family containing a common structural feature and conserved residues for catalysis and substrate binding [36,39,43]. From the 3D model of TreS, we speculated that the deep groove in the catalytic pocket served as an entry point of substrate into the catalytic center and as an export site of products (Figure 5B). Our study showed that residues of catalytic importance in GH13 family enzymes were also conserved in TreS (Figure 1), and the importance of the identified five residues in the central catalytic area (His172, Asp201, Glu251, His318 and Asp319) was revealed in a mutagenesis study (Table 3). The same conserved amino acids were found in other similar class of GH13 family enzymes, such as *Bacillus cereus* oligo-1,6-glucosidase (His103, Asp199, Glu255, His328, and Asp329) [44], *Neisseria polysacchareais* amylosucrase (His187, Asp286, Glu328, His392, and Asp393) [45], and *Pseudomonas mesoacidophila* MX-45 trehalulose synthase (His104, Asp200, Glu254, His326, and Asp327) [46]. This finding is consistent with previous suggestions and further supported the assumption that TreS employs a similar hydrolysis mechanism as other GH13 family enzymes.

In conclusion, construction and screening of a large-insert soil-derived metagenomic library has led to the discovery and characterization of one novel trehalose synthase. The novelty of this enzyme arises from the enormous genetic diversity of uncultured saline-alkali soil microorganisms. The recovered enzyme displayed several excellent enzymatic properties, such as resistance to most metal ions, high activity over a wide range of temperatures and pH values, with the maximum trehalose yield to be above 78%, which was considered as a good candidate for the large-scale production of trehalose in the near future. 

## References

[1] Schiraldi C, Di Lernia I, De Rosa M (2002) Trehalose production: exploiting novel approaches. Trends Biotechnol 20: 420-425. doi:10.1016/S0167-7799(02)02041-3. PubMed: 12220904. 12220904

[2] Elbein AD, Pan YT, Pastuszak I, Carroll D (2003) New insights on trehalose: a multifunctional molecule. Glycobiology 13: 17R-27R. doi:10.1093/glycob/cwg047. PubMed: 12626396. 12626396

[3] Zhang R, Pan YT, He SM, Lam M, Brayer GD et al. (2001) Mechanistic analysis of trehalose synthase from *Mycobacterium* *smegmatis* . J Biol Chem 286: 35601-35609. PubMed: 21840994. 10.1074/jbc.M111.280362 PMC3195595 21840994

[4] Kandror O, DeLeon A, Goldberg AL (2002) Trehalose synthesis is induced upon exposure of *Escherichia* *coli* to cold and is essential for viability at low temperatures. Proc Natl Acad Sci U S A 99: 9727-9732. doi:10.1073/pnas.142314099. PubMed: 12105274. 12105274 PMC124994

[5] Purvis JE, Yomano LP, Ingram LO (2005) Enhanced trehalose production improves growth of *Escherichia* *coli* under osmotic stress. Appl Environ Microbiol 71: 3761-3769. doi:10.1128/AEM.71.7.3761-3769.2005. PubMed: 16000787. 16000787 PMC1168978

[6] Gancedo C, Flores CL (2004) The importance of a functional trehalose biosynthetic pathway for the life of yeasts and fungi. FEMS Yeast Res 4: 351-359. doi:10.1016/S1567-1356(03)00222-8. PubMed: 14734015. 14734015

[7] Gélinas P, Fiset G, Leduy A, Goulet J (1989) Effect of growth-conditions and trehalose content on cryotolerance of bakers’ yeast in frozen doughs. Appl Environ Microbiol 55: 2453-2459. PubMed: 16348024. 16348024 10.1128/aem.55.10.2453-2459.1989 PMC203104

[8] Crowe JH, Hoekstra FA, Crowe LM (1992) Anhydrobiosis. Annu Rev Physiol 54: 579-599. doi:10.1146/annurev.ph.54.030192.003051. PubMed: 1562184. 1562184

[9] Grba S, Oura E, Suomalainen H (1975) On the formation of glycogen and trehalose in baker’s yeast. Eur J Appl Microbiol 2: 29-31. doi:10.1007/BF01385443.

[10] Hottiger T, Devirgilio C, Hall MN, Boller T, Wiemken A (1994) The role of trehalose synthesis for the acquisition of thermotolerance in yeast. Eur J Biochem 19: 187-193. 10.1111/j.1432-1033.1994.tb19929.x 8306985

[11] Conlin LK, Nelson HCM (2007) The natural osmolyte trehalose is a positive regulator of the heat-induced activity of yeast heat shock transcription factor. Mol Cell Biol 27: 1505-1515. doi:10.1128/MCB.01158-06. PubMed: 17145780. 17145780 PMC1800720

[12] Jain NK, Roy I (2009) Effect of trehalose on protein structure. Protein Sci 18: 24-36. PubMed: 19177348. 19177348 10.1002/pro.3 PMC2708026

[13] Benaroudj N, Lee DH, Goldberg AL (2001) Trehalose accumulation during cellular stress protects cells and cellular proteins from damage by oxygen radicals. J Biol Chem 276: 24261-24267. doi:10.1074/jbc.M101487200. PubMed: 11301331. 11301331

[14] Ohtake S, Wang YJ (2011) Trehalose: current use and future applications. J Pharm Sci 100: 2020-2053. doi:10.1002/jps.22458. PubMed: 21337544. 21337544

[15] Hunter RL, Armitige L, Jagannath C, Actor JK (2009) TB Research at UT-Houston-A review of cord factor: new approaches to drugs, vaccines and the pathogenesis of tuberculosis. Tuberculosis 89: S18-S25. doi:10.1016/S1472-9792(09)70007-1. PubMed: 20006299. 20006299 PMC3682682

[16] Hunter RL, Olsen MR, Jagannath C, Actor JK (2006) Multiple roles of cord factor in the pathogenesis of primary, secondary, and cavitary tuberculosis, including a revised description of the pathology of secondary disease. Ann Clin Lab Sci 36: 371-386. PubMed: 17127724. 17127724

[17] Paiva CL, Panek AD (1996) Biotechnological applications of the disaccharide trehalose. Biotechnol Annu Rev 2: 293-314. doi:10.1016/S1387-2656(08)70015-2. PubMed: 9704101. 9704101

[18] Maruta K, Nakada T, Kubota M, Chaen H, Sugimoto T et al. (1995) Formation of trehalose from maltooligosaccharides by a novel enzymatic system. Biosci Biotechnol Biochem 59: 1829-1834. doi:10.1271/bbb.59.1829. PubMed: 8534970. 8534970

[19] Chang SW, Liu PT, Hsu LC, Chen CS, Shaw JF (2012) Integrated biocatalytic process for trehalose production and separation from rice hydrolysate using a bioreactor system. Food Chem 134: 1745-1753. doi:10.1016/j.foodchem.2012.03.065. PubMed: 23442616. 23442616

[20] Nishimoto T, Nakano M, Ikegami S, Chaen H, Fukuda S et al. (1995) Existence of a novel enzyme converting maltose into trehalose. Biosci Biotechnol Biochem 59: 2189-2190. doi:10.1271/bbb.59.2189.

[21] Schloss PD, Handelsman J (2005) Metagenomics for studying unculturable microorganisms: cutting the Gordian knot. Genome Biol 6: 229. doi:10.1186/gb-2005-6-8-229. PubMed: 16086859. 16086859 PMC1273625

[22] Benoliel B, Poças-Fonseca MJ, Torres FAG, de Moraes LMP (2010) Expression of a Glucose-tolerant beta-glucosidase from *Humicola* *grisea* var. thermoidea in *Saccharomyces* *cerevisiae* . Appl Biochem Biotechnol 160: 2036-2044. doi:10.1007/s12010-009-8732-7. PubMed: 19669941. 19669941

[23] Simon C, Daniel R (2009) Achievements and new knowledge unraveled by metagenomic approaches. Appl Microbiol Biotechnol 85: 265-276. doi:10.1007/s00253-009-2233-z. PubMed: 19760178. 19760178 PMC2773367

[24] Yao J, Fan XJ, Lu Y, Liu YH (2011) Isolation and characterization of a novel tannase from a metagenomic library. J Agric Food Chem 59: 3812-3818. doi:10.1021/jf104394m. PubMed: 21388130. 21388130

[25] Zhou JZ, Bruns MA, Tiedje JM (1996) DNA recovery from soils of diverse composition. Appl Environ Microbiol 62: 316-322. PubMed: 8593035. 8593035 10.1128/aem.62.2.316-322.1996 PMC167800

[26] Liang JY, Huang RB, Huang Y, Wang XB, Du LQ et al. (2013) Cloning, expression, properties, and functional amino acid residues of new trehalose synthase from *Thermomonospora* *curvata* DSM 43183. J Mol Catal Benzym 90: 26-32. doi:10.1016/j.molcatb.2013.01.014.

[27] Jiang L, Li S, Hu Y, Xu Q, Huang H (2012) Adaptive evolution for fast growth on glucose and the effects on the regulation of glucose transport system in *Clostridium* *tyrobutyricum* . Biotechnol Bioeng 109(3): 708-718. doi:10.1002/bit.23346. PubMed: 21956266. 21956266

[28] Guex N, Peitsch MC (1997) SWISS-MODEL and the Swiss-Pdb Viewer: An environment for comparative protein modeling. Electrophoresis 18: 2714- 2723. doi:10.1002/elps.1150181505. PubMed: 9504803. 9504803

[29] Filipkowski P, Pietrow O, Panek A, Synowiecki J (2012) Properties of recombinant trehalose synthase from *Deinococcus* *radiodurans* expressed in *Escherichia* *coli* . Acta Biochim Pol 59: 425-431. PubMed: 23032750. 23032750

[30] Van Vaeck C, Wera S, Van Dijck P, Thevelein JM (2001) Analysis and modification of trehalose 6-phosphate levels in the yeast *Saccharomyces* *cerevisiae* with the use of *Bacillus* *subtilis* phosphotrehalase. Biochem J 353: 157-162. PubMed: 11115409. 11115409 PMC1221553

[31] Woo EJ, Ryu SI, Song HN, Jung TY, Yeon SM et al. (2010) Structural insights on the new mechanism of trehalose synthesis by trehalose synthase TreT from *Pyrococcus* *horikoshii* . J Mol Biol 404: 247-259. doi:10.1016/j.jmb.2010.09.056. PubMed: 20888836. 20888836

[32] Han SE, Kwon HB, Lee SB, Yi BY, Murayama I et al. (2003) Cloning and characterization of a gene encoding trehalose phosphorylase (TP) from *Pleurotus* *sajor-caju* . Protein Expr Purif 30: 194-202. doi:10.1016/S1046-5928(03)00104-9. PubMed: 12880768. 12880768

[33] Kim YH, Kwon TK, Park S, Seo HS, Cheong JJ et al. (2000) Trehalose synthesis by sequential reactions of recombinant maltooligosyltrehalose synthase and maltooligosyltrehalose trehalohydrolase from *Brevibacterium* *helvolum* . Appl Environ Microbiol 66: 4620-4624. doi:10.1128/AEM.66.11.4620-4624.2000. PubMed: 11055902. 11055902 PMC92358

[34] Avonce N, Mendoza-Vargas A, Morett E, Iturriaga G (2006) Insights on the evolution of trehalose biosynthesis. BMC Evol Biol 6: 109. doi:10.1186/1471-2148-6-109. PubMed: 17178000. 17178000 PMC1769515

[35] Wei YT, Zhu QX, Luo ZF, Lu FS, Chen FZ et al. (2004) Cloning, expression and identification of a new trehalose synthase gene from *Thermobifida* *fusca* genome. Acta Biochim Biophys Sin 36: 477-484. doi:10.1093/abbs/36.7.477. PubMed: 15248022. 15248022

[36] Yue M, Wu XL, Gong WN, Ding HB (2009) Molecular cloning and expression of a novel trehalose synthase gene from *Enterobacter* *hormaechei* . Microb Cell Factories 8: 34. doi:10.1186/1475-2859-8-34. PubMed: 19523196. PMC2701924 19523196

[37] Wu XL, Ding HB, Yue M, Qiao Y (2009) Gene cloning, expression, and characterization of a novel trehalose synthase from *Arthrobacter* *aurescens* . Appl Microbiol Biotechnol 83: 477-482. doi:10.1007/s00253-009-1863-5. PubMed: 19172263. 19172263

[38] Buisson G, Duée E, Haser R, Payan F (1987) Three dimensional structure of porcine pancreatic alpha-amylase at 2.9 A resolution. Role of calcium in structure and activity. EMBO J 6: 3909-3916. PubMed: 3502087. 3502087 10.1002/j.1460-2075.1987.tb02731.x PMC553868

[39] Chen YS, Lee GC, Shaw JF (2006) Gene cloning, expression, and biochemical characterization of a recombinant trehalose synthase from *Picrophilus* *torridus* in *Escherichia* *coli* . J Agric Food Chem 54: 7098-7104. doi:10.1021/jf060828q. PubMed: 16968068. 16968068

[40] Wang JH, Tsai MY, Chen JJ, Lee GC, Shaw JF (2007) Role of the C-terminal domain of *Thermus* *thermophilus* trehalose synthase in the thermophilicity, thermostability, and efficient production of trehalose. J Agric Food Chem 55: 3435-3443. doi:10.1021/jf070181p. PubMed: 17394343. 17394343

[41] Lee JH, Lee KH, Kim CG, Lee SY, Kim GJ et al. (2005) Cloning and expression of a trehalose synthase from *Pseudomonas* *stutzeri* CJ38 in *Escherichia* *coli* for the production of trehalose. Appl Microbiol Biotechnol 68: 213-219. doi:10.1007/s00253-004-1862-5. PubMed: 15654636. 15654636

[42] Tewari YB, Goldberg RN (1991) Thermodynamics of hydrolysis of disaccharides. Lactulose, alpha-D-melibiose, palatinose, D-trehalose, D-turanose and 3-o-beta-D-galactopyranosyl-D-arabinose. Biophys Chem 40: 59-67. doi:10.1016/0301-4622(91)85029-P. PubMed: 1873472. 1873472

[43] Cantarel BL, Coutinho PM, Rancurel C, Bernard T, Lombard V et al. (2009) The Carbohydrate-Active EnZymes database (CAZy): an expert resource for glycogenomics. Nucleic Acids Res 37: D233-D238. doi:10.1093/nar/gkn663. PubMed: 18838391. 18838391 PMC2686590

[44] Watanabe K, Hata Y, Kizaki H, Katsube Y, Suzuki Y (1997) The refined crystal structure of *Bacillus* *cereus* oligo-1,6-glucosidase at 2.0 angstrom resolution: Structural characterization of proline-substitution sites for protein thermostabilization. J Mol Biol 269: 142-153. doi:10.1006/jmbi.1997.1018. PubMed: 9193006. 9193006

[45] Mirza O, Skov LK, Remaud-Simeon M, de Montalk GP, Albenne C et al. (2001) Crystal structures of amylosucrase from *Neisseria* *polysaccharea* in complex with D-glucose and the active site mutant Glu328Gln in complex with the natural substrate sucrose. Biochemistry 40: 9032-9039. doi:10.1021/bi010706l. PubMed: 11467966. 11467966

[46] Ravaud S, Robert X, Watzlawick H, Haser R, Mattes R et al. (2007) Trehalulose synthase native and carbohydrate complexed structures provide insights into sucrose isomerization. J Biol Chem 282: 28126-28136. doi:10.1074/jbc.M704515200. PubMed: 17597061. 17597061

